# Reducing Viability Bias in Analysis of Gut Microbiota in Preterm Infants at Risk of NEC and Sepsis

**DOI:** 10.3389/fcimb.2017.00237

**Published:** 2017-06-06

**Authors:** Gregory R. Young, Darren L. Smith, Nicholas D. Embleton, Janet E. Berrington, Edward C. Schwalbe, Stephen P. Cummings, Christopher J. van der Gast, Clare Lanyon

**Affiliations:** ^1^Faculty of Health and Life Sciences, University of NorthumbriaNewcastle upon Tyne, United Kingdom; ^2^Newcastle Neonatal Service, Newcastle upon Tyne Hospitals NHS Foundation TrustNewcastle upon Tyne, United Kingdom; ^3^School of Science and Engineering, Teesside UniversityMiddlesbrough, United Kingdom; ^4^School of Healthcare Science, Manchester Metropolitan UniversityManchester, United Kingdom

**Keywords:** preterm, neonate, stool, microbiota, viability, propidium monoazide

## Abstract

Necrotising enterocolitis (NEC) and sepsis are serious diseases of preterm infants that can result in feeding intolerance, the need for bowel resection, impaired physiological and neurological development, and high mortality rates. Neonatal healthcare improvements have allowed greater survival rates in preterm infants leading to increased numbers at risk of developing NEC and sepsis. Gut bacteria play a role in protection from or propensity to these conditions and have therefore, been studied extensively using targeted 16S rRNA gene sequencing methods. However, exact epidemiology of these conditions remain unknown and the role of the gut microbiota in NEC remains enigmatic. Many studies have confounding variables such as differing clinical intervention strategies or major methodological issues such as the inability of 16S rRNA gene sequencing methods to determine viable from non-viable taxa. Identification of viable community members is important to identify links between the microbiota and disease in the highly unstable preterm infant gut. This is especially important as remnant DNA is robust and persists in the sampling environment following cell death. Chelation of such DNA prevents downstream amplification and inclusion in microbiota characterisation. This study validates use of propidium monoazide (PMA), a DNA chelating agent that is excluded by an undamaged bacterial membrane, to reduce bias associated with 16S rRNA gene analysis of clinical stool samples. We aim to improve identification of the viable microbiota in order to increase the accuracy of clinical inferences made regarding the impact of the preterm gut microbiota on health and disease. Gut microbiota analysis was completed on stools from matched twins (*n* = 16) that received probiotics. Samples were treated with PMA, prior to bacterial DNA extraction. Meta-analysis highlighted a significant reduction in bacterial diversity in 68.8% of PMA treated samples as well as significantly reduced overall rare taxa abundance. Importantly, overall abundances of genera associated with protection from and propensity to NEC and sepsis such as: *Bifidobacterium; Clostridium*, and *Staphylococcus* sp. were significantly different following PMA-treatment. These results suggest non-viable cell exclusion by PMA-treatment reduces bias in gut microbiota analysis from which clinical inferences regarding patient susceptibility to NEC and sepsis are made.

## Introduction

Severely preterm infants (<32 weeks) have immature immune systems (Levy, 2007; Strunk et al., 2011), improperly formed intestinal lumen (Halpern and Denning, 2015), and often feeding intolerance (Fanaro, 2013). All such characteristics increase the risk of onset of nosocomial infection, necrotising enterocolitis (NEC), and sepsis (Gregory et al., 2011). Outbreaks of NEC within the neonatal intensive care unit (NICU) (Boccia et al., 2001) and the absence of such diseases prior to bacterial colonization at birth suggest a key role of gut bacterial dysbiosis in these conditions. True causation is, however, very difficult to identify and compacted by complex to understand, highly turbulent community characteristics in the gut, probably in part affected by “routine” interventions of neonatal intensive care (antibiotic administration, feeding strategies, etc.) (Stoll et al., 1996; Hoy et al., 2000).

Targeted 16S rRNA gene sequencing technologies are used to produce microbial metadata for entire populations of a biotope with superior depth, specificity and, most importantly, in significantly less time than previous culture-based or molecular methods (Weinstock, 2012). In addition, the price of sequencing continues to decline (Caporaso et al., 2012), providing further incentives for microbiologists to employ this technique. However, 16S rRNA gene sequencing does introduce inherent biases (von Wintzingerode et al., 1997), including enrichment of particular bacterial groups before storage (Rochelle et al., 1994), insufficient or preferential disruption of certain bacterial cells (Leff et al., 1995; Schneegurt et al., 2003), introduction of sequencing artefacts such as chimeras (Wang and Wang, 1996) and inability to exclude DNA from non-viable sources (Nocker et al., 2006; Nocker and Camper, 2009; Rogers et al., 2013). Persistence of non-viable DNA is due to the stability of the molecule, which enables DNA to remain in an environment long after the originating organism has died. DNA from non-viable bacterial cells (NVBCs) can persist in the lumen of the GI tract, resulting in identification during targeted 16S rRNA gene sequencing analyses. Such bias is especially important whilst studying the highly unstable (Koenig et al., 2011; Bergstrom et al., 2014), low diversity (Tuddenham and Sears, 2015) gut bacterial communities of severely preterm infants. A technique to enable non-viable cell exclusion (NVCE), from such analyses is, therefore, an important and necessary requirement in order to reduce bias and improve the current understanding of bacterial taxa associated with NEC and sepsis.

PMA is a DNA chelating compound that cannot translocate across a viable cellular membrane (Nocker et al., 2007). Nocker et al. (2006), developed the use of Propidium Monoazide (PMA), for differentiation between viable and non-viable bacterial cells during targeted 16S rRNA gene sequencing microbiota analyses (Nocker et al., 2006; Nocker and Camper, 2009; Rogers et al., 2013). This process has been applied in microbial ecology studies of other environments, including wastewater samples (Nocker et al., 2007, 2010), human oral cavities (Sanchez et al., 2013), human adult faeces (Bae and Wuertz, 2009; Fujimoto and Watanabe, 2013), the cystic fibrosis lung (Rogers et al., 2010; Nguyen et al., 2016), and other lower lung respiratory infections (Rogers et al., 2013). The technique, however, has not yet been validated for use in the unique biotope of preterm infant stool despite vast quantities of research being published regarding this microbiota (Mshvildadze et al., 2010; Mai et al., 2011; Torrazza et al., 2013; McMurty et al., 2015). Furthermore, no studies so far have validated combining PMA treatment of this sample type in conjunction with the Schloss method for paired end targeted 16S rRNA gene sequencing (Kozich et al., 2013).

This study aims to identify and alleviate the bias associated with non-viable bacterial DNA inclusion in studies of the gut microbiota of significantly preterm infants at risk of NEC and sepsis. In doing so we hope to increase the accuracy of microbiota characterization in patients at risk of NEC and sepsis, therefore improving the quality of clinical inferences made in relation to the conditions.

The effects of PMA treatment were assessed by comparing bacterial richness, diversity, and community structure as well as individual taxa abundances within PMA-treated and untreated frozen stool samples (*n* = 16) when assessed using targeted paired end sequencing of the 16S rRNA gene.

## Materials and methods

Faecal samples were collected when available from day of life 43–81 from a set of significantly preterm twins born 25(+2) weeks gestation and at ≤710 g, enrolled on the SERVIS study at the Royal Victoria Infirmary NICU, Newcastle upon Tyne, England, with ethical permission (NRES Committee North East—Newcastle & North Tyneside 2). Both patients were administered Infloran® (Laboratorio Farmaceutico SIT, Mede, PV, ITA) probiotic supplements throughout the course of the sampling period (*Bifidobacterium bifidum, Lactobacillus acidophilus*). Stool was collected in sterile glass pots with sealed lids and frozen immediately on the ward. Batch collection and transportation to freezers at Northumbria University followed. Samples were stored at −80°C until PMA treatment and DNA extraction for analysis.

### PMA treatment and DNA extraction

PMA was supplied by Biotium (Hayward, CA, USA), and dissolved in dimethyl sulfoxide to a stock concentration of 20 mM. Faecal samples were homogenised in 2.5 ml PBS per 0.1 g of stool (≤0.5 g), and centrifuged. The centrifuged pellet was resusupended in 2 ml PBS and split evenly to facilitate PMA-treated and untreated conditions per sample. PMA stock solution was added to a final concentration of 50 μM in treated samples and the equivalent volume of PBS was added to untreated samples. PMA cross-linking was initiated by 30 min incubation on ice, in the dark with occasional mixing. Following this, samples were exposed to blue LED light at 464 nm during 30-s intervals for a total of 2 min. After light exposure, samples were centrifuged at 10,000 × g for 5 min. The supernatant was discarded and DNA extracted from the cellular pellet using MoBio PowerLyzer PowerSoil DNA Isolation Kit (Carlsbad, CA, USA), as per manufacturer's instructions.

### Nested PCR protocol and MiSeq analysis

Prior to paired end targeted 16S rRNA gene analysis, extracted viable DNA was amplified by PCR. Nested PCR was employed in this scenario not to increase copy number prior to sequencing but to increase impact of PMA-intercalation of DNA by blocking amplification of the whole 16S rRNA gene sequence prior to targeted sequencing of the shorter V4 region. Banihashemi et al. (2012) showed that amplification of a 200 bp fragment failed to omit dead cell signals fully from DNA based community analyses. Universal bacterial 16S rRNA gene specific primers 27f (Lane, 1991), and 1,492r (Turner et al., 1999) were used under the following conditions: initial denaturation at 95°C for 5 min then 25 cycles of 30 s denaturation at 95°C; primer annealing at 44.5°C for 30 s; elongation at 72°C for 30 s then a final elongation at 72°C for 10 min.

PCR products were serially diluted 1:10 and paired end targeted analysis of V4 regions of the 16S rRNA gene was performed as described by Kozich et al. (2013), on the Illumina MiSeq using primers described by Caporaso et al. (2011). MiSeq 250 × 2 chemistry was used to perform the targeted 16S rRNA sequencing.

### Analysis

Sequence reads with phred-score ≥Q30 were trimmed, merged and processed in Mothur (Schloss et al., 2009), following the MiSeq SOP. Number of sequences passing Q30 in each sample are illustrated in Figure S1. Reads with phred-score <Q30 were not included in analysis. Uncorrected pairwise distances were calculated before clustering sequences in to OTUs using average neighbor joining, as recommended by Schloss and Westcott (2011). The same sequence reads were also submitted to the EBI ENA database for analysis (study accession PRJEB10326; http://www.ebi.ac.uk/ena/data/view/PRJEB10326).

Singletons were not removed from analysis to allow identification of PMA-treatment on all rare taxa identified by targeted sequencing. Normalization was not performed by rarefaction or subsampling due to the nature of the investigation. Instead relative abundances of individual taxa per sample were calculated. This is because the impact of PMA NVCE was assessed by omission of sequence reads from the community, therefore the absence of any sequence read was as informative as the presence of the same.

Per sample richness and beta-diversity was calculated using R statistical software (R_Core_Team., 2014) and the vegan package for community ecology (Oksanen et al., 2015). Meta-analysis (Borenstein et al., 2009) was used to compare results by treatment condition. Meta-analysis has previously been used to quantify the effect of PMA-treatment on bacterial communities of expectorated CF sputum samples (Rogers et al., 2013), allowing direct comparison of the effect of PMA-treatment between paired and unpaired samples by comparing effect size, rather than comparing means of highly variable individual samples by *t*-test. Each microbiota was randomly sub-sampled with bootstrapping *n* = 1,000 times. Standard error was reported.

SIMPER comparison of individual taxa relative abundance per treatment condition was performed using PAST (Hammer et al., 2001). Significance of results was calculated and plotted using R statistical software.

Comparison of non-frozen and frozen stool microbiotas was performed using ANOSIM and unconstrained Morisita–Horn cluster analysis.

## Results

Stool samples from a set of significantly preterm twins (25+2 weeks gestation) (*n* = 16) receiving Infloran® probiotic supplements were subjected to PMA-treatment for comparison to an untreated control of each sample. 16S rRNA gene sequencing identified a total of 161 individual taxa producing 4.72 × 10^6^ total reads from 16 × 2 samples.

### Identification of common and rare taxa

**Figure 1:**
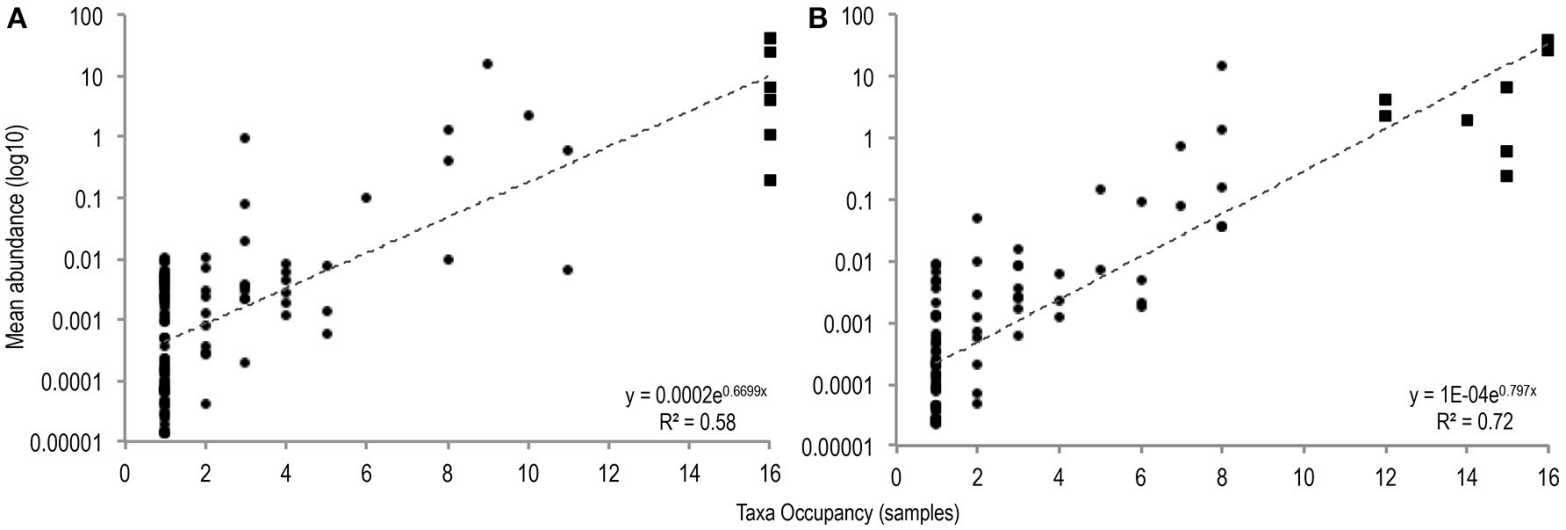
The number of samples in which taxa were observed plotted against mean taxa abundance (log10 scale) in untreated sample **(A)**, and PMA-treated sample **(B)**, conditions [(**A**: *r*^2^ = 0.58; *P* = <0.001), (**B**: *r*^2^ = 0.72; *P* = < 0.001)]. Common taxa (>75% sample occupancy) are plotted square, rare samples (<75% sample occupancy) are plotted circle.

To identify differences between common and rare taxa in PMA-treated and untreated conditions distribution abundance relationship plots were produced (Figure 1).

Significant positive distribution abundance relationships were observed between taxa abundance and persistence of taxa across samples in both treatment conditions (untreated: *r*^2^ = 0.58, *n* = 120, *P* = < 0.001; PMA-treated: *r*^2^ = 0.72, *n* = 97, *P* = < 0.001). Using this relationship, taxa in the upper quartile of occupancy (≥75% samples), in each treatment condition were classified as common, the remaining taxa were classified as rare.

Distribution of taxa appeared more even in the PMA-treated condition: fewer ubiquitous taxa dominate the communities in the PMA-treated condition (2 taxa); compared to the untreated condition (6 taxa).

In untreated sample conditions 6 taxa were identified as common, all of which were observed in every sample. *Bifidobacterium, Enterococcus*, 2 *Clostridia* spp., a *Veillonella* and an unclassified Enterobacteriaceae accounted for 77.9% of the total community member sequences. In PMA-treated samples, 8 common taxa were identified, comprising 82.2% of total community member sequences however of these, only 2 (*Bifidobacterium* and *Enterococcus*) were found in all samples. *Anaerococcus* and *Finegoldia* sp. were identified as common in PMA-treated samples but not in untreated samples.

### Effect of PMA treatment on bacterial richness and diversity

Due to the large coverage variability between the stool sample communities (*m* = 1.47 × 10^5^, *SD* = 1.26 × 10^5^), meta-analysis was used to identify the effect size of PMA-treatment on microbiota composition by bacterial OTU richness (O*); Shannon diversity index (H′); and Inverse Simpson's diversity index (1/D).

**Figure 2:**
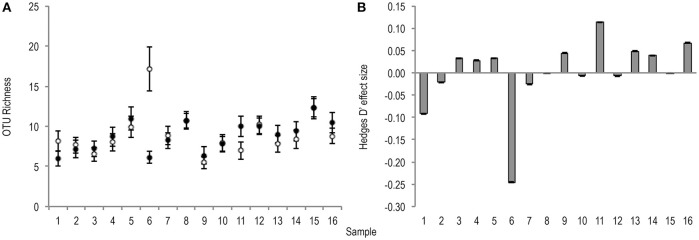
**(A)** Bootstrapped (*n* = 1,000) O* values for untreated (hollow points), and PMA-treated (solid points) conditions of all samples (1–16). Error bars are included. **(B)** Hedges' d effect size of PMA-treatment on O* of all samples (1–16). Positive effect was observed in 50% of samples. Sample 6 was the only observed effect size >0.2 (small effect).

Bacterial OTU richness was variable between stool samples of the same treatment condition (untreated *m* = 9.1 ± 2.7, PMA-treated *m* = 8.8 ± 1.9). The effect of PMA-treatment on O* was only once greater than the significance threshold (0.2), and showed no directional consistency (Figure 2).

Like richness, bacterial diversity also varied between individual stool samples in the same treatment condition (Table 1).

**Table 1:** Mean and standard deviation for Shannon and inverse Simpson diversity indices for PMA-treated and untreated conditions.

	**Diversity index**			
	**Shannon (H')**		**Inverse Simpson (1/D)**	
	**Mean**	***SD***	**Mean**	***SD***
PMA-treated	1.13	0.32	2.64	0.95
Non-PMA-treated	1.30	0.16	2.99	0.50
*P*-value	<0.05		>0.05	

**Figure 3:**
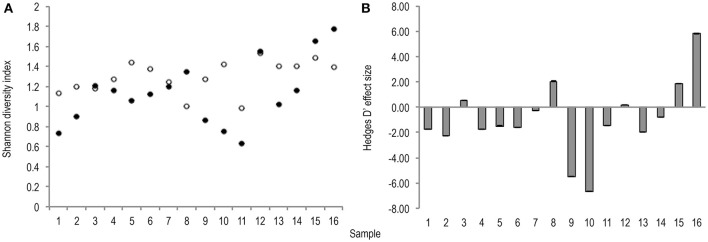
**(A)** Bootstrapped (*n* = 1,000) Shannon diversity index values for untreated (hollow points), and PMA-treated (solid points), conditions for all samples (1–16). Standard error bars are included. **(B)** Hedges' d effect size of PMA-treatment on Shannon diversity index values of all samples (1–16). Seventy-five percentage of samples exhibit significant (>0.8) effect size, 75% of which are negative.

**Figure 4:**
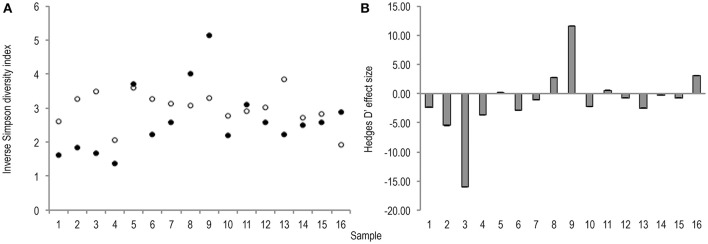
**(A)** Bootstrapped (*n* = 1,000), inverse Simpson diversity index values for untreated (hollow points), and PMA-treated (solid points), conditions for all samples (1–16). Standard error bars are included but error is too insignificant to be visible. **(B)** Hedges' d effect size of PMA-treatment on inverse Simpson diversity index values of all samples (1–16). 68.8% samples exhibit significant (>0.8) effect size. 72.3% of which are negative rare **(B)** taxa.

Meta-analysis showed negative effect sizes of PMA-treatment on bacterial diversity in 71.9% of samples, of which 73.9% were highly significant (>0.8) (Figures 3, 4). Significant negative mean overall effect sizes on both measures of diversity were observed following PMA-treatment (m: H′ = −0.95; 1/D = −1.23).

### Effect of PMA-treatment on individual bacterial taxa abundance

To investigate the effect of PMA-treatment on observable abundance values of individual taxa relative sequence abundance was calculated. Initial analysis of the PMA-treatment effect on individual taxa abundance within the bacterial communities of stools was performed by SIMPER (Table 2). SIMPER provides an insight in to the variance, expressed as a percentage, between abundance of taxa from the untreated group and the PMA-treated group.

**Table 2:** SIMPER analysis of common **(A)** and rare **(B)** taxa.

**Average dissimilarity between conditions** = **42.87**				**Non-PMA-treated**	**PMA-treated**
**Taxon**	**Av. dissim**	**Contrib. %**	**Cumulative %**	**Mean abund. 1**	**Mean abund. 2**
**(A)**					
*Bifidobacterium*	15.20	35.44	35.44	41.1	26.5
*Enterococcus*	12.23	28.52	63.96	25.0	39.8
*Clostridium 1*	4.60	10.74	74.70	4.0	6.6
Enterobacteriaceae; unclassified	4.03	9.41	84.11	6.6	0.6
Anaerococcus^*^	3.25	7.58	91.68	2.2	4.2
Finegoldia^*^	1.75	4.08	95.76	1.3	2.3
*Clostridium 2*	1.08	2.51	98.27	0.2	1.9
*Veillonella*	0.74	1.73	100.00	1.1	0.2
**Average dissimilarity between conditions** = **83.21**				**Non-PMA-treated**	**PMA-treated**
**(B)**					
*Escherichia_Shigella*	52.81	63.46	63.46	16.2	14.9
*Peptoniphilus*	11.07	13.3	76.76	0.576	1.41
*Actinomyces*	6.88	8.267	85.03	0.389	0.75
*Streptococcus*	4.087	4.911	89.94	0.986	0.0157
*Staphylococcus*	1.378	1.656	91.59	0	0.15
*Phenylobacterium*	1.333	1.602	93.2	0	0.0939

*Cut-off value set at taxa contributing < 1% to dissimilarity between treatment conditions*.

* *Denotes taxa only attributed common status in PMA-treated condition*.

*Cut-off set at taxa contributing < 1% to dissimilarity between treatment conditions*.

Table 2 illustrates which taxa contributed greatest to dissimilarity of common and rare community structures between untreated and PMA-treated conditions.

Greater average dissimilarity is observed in rare (83.21%), than common (42.87), taxa. The taxon labelled *Escherichia_shigella* by the *SILVA* database (Quast et al., 2013) appears to contribute to the average dissimilarity between non-PMA and PMA treated conditions most (63.45%), in spite of a mean abundance difference of only 1.3%. This contrasts with other taxa such as *Bifidobacterium* and *Enterococcus*, for which lower average dissimilarities of 15.20 and 12.25 are observed, however much greater mean abundance differences of 14.6 and 14.8 are found, respectively. This incongruence could be explained by variation in abundance of individual taxa between samples within the same condition. The SD of *Escherichia* abundance is much greater (untreated: *m* = 16.2, *SD* = 18.2; PMA-treated: *m* = 14.9, *SD* = 24.3), than that of *Bifidobacterium* (untreated: *m* = 41.1, *SD* = 15.2; PMA-treated: *m* = 26.5, *SD* = 19.5), or *Enterococcus* (untreated: *m* = 25.0, *SD* = 10.2; PMA-treated: *m* = 39.8, *SD* = 14.0).

**Figure 5:**
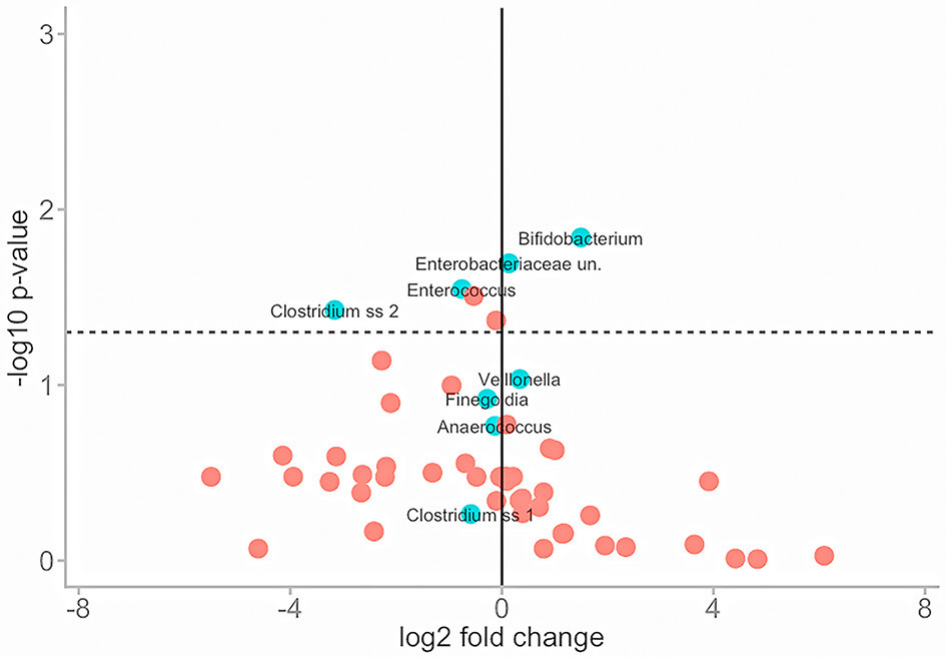
Illustrates the effect of PMA-treatment on individual taxa abundances. Fold change in abundance is plotted (*x*) against the *P*-value of that same fold change (*y*) for each taxon. The solid vertical line represents no fold-change. Travelling in either direction from this line the fold change increases. The dotted horizontal line represents significance cut-off (.05). Core taxa are blue and labelled. Rare taxa are red.

To normalise for this variance Wilcoxon rank sum tests were performed to assess the similarities of mean abundance for each taxon in both conditions as identified by SIMPER analysis. The same means were used to calculate a fold change in taxa abundance between the two conditions and both parameters were plotted on a volcano plot (Figure 5).

The majority of taxa showed substantial fold changes in abundance following PMA-treatment; however only 6 of these fold changes pass the significance threshold (*P* < 0.05): *Bifidobacterium*;Enterobacteriaceae;*Enterococcus; Clostridium; Actinomyces;* and *Peptoniphilus* sp.

In untreated conditions *Bifidobacterium* and *Enterobacteriaceae* sp. abundances are significantly greater while *Enterococcus, Clostridium, Peptoniphilus*, and *Actinomyces* sp. abundances are significantly lower. This suggests that the presence of non-viable DNA originating from highly abundant species such as *Bifidobacteria* and *Enterobacteriaceae* could potentially mask that of less abundant species such as *Enterococcus, Clostridium, Peptoniphilus*, and *Actinomyces* sp.

**Figure 6:**
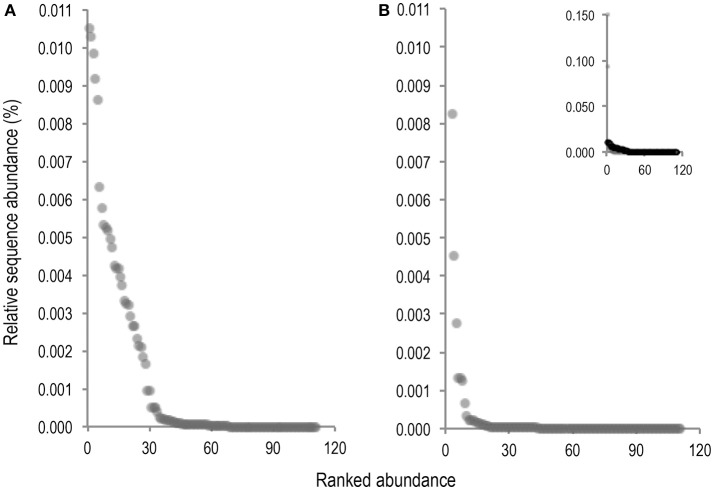
Rank abundance plots of taxa for which abundance was only measurable in either the untreated **(A)**, or PMA-treated **(B)**, sample condition. Ranked abundance of the untreated condition including *Staphylococcus* and *Phenylobacterium* is inset at top right corner of PMA-treated condition plot **(B)**.

As volcano plots (Figure 5) only represent fold change in abundance for taxa present in both sample conditions, rank abundance plots (Figure 6) were generated to illustrate abundance of taxa identifiable in only one treatment condition.

Fewer taxa were observed PMA-treated than untreated sample conditions. Of 111 total taxa present in only one condition 68 (61.3%), were present in untreated samples while only 43 (38.7%), were present in PMA-treated samples, representing a 22.6% reduction in presence of taxa measurable in only one condition. Levels of 2 rare taxa (*Staphylococcus* and *Phenylobacterium*), were observable at levels > 0.09% sequence abundance (almost 10 fold more than all other taxa observable in only one treatment condition), following PMA-treatment. These taxa were completely masked in the untreated condition. All other taxa abundances were reduced to <0.009% following PMA-treatment. A significant difference between mean abundances of rare taxa presence between untreated (*m* = 0.0052), and PMA-treated (*m* = 0.0012), sample conditions (*P* = <0.001), was observed. This suggests that performing gut microbiota analysis of frozen stool by paired end targeted 16S rRNA gene sequencing without PMA-treatment could fail to identify presence of rare community members due to significant background sequence noise origniating from non-living taxa. This could explain the significant overall reduction in mean bacterial diversity following PMA-treatment observed in meta-analysis (Figures 3, 4). Principle coordinate analysis of sample communities was also performed based on Bray-Curtis dissimilarity of taxa abundances (Figure S2).

To confirm the differences observed were due to genuine viable differences in the sample microbiota rather than bacterial cell death during freezing (−80°C) one further stool sample was split (*n* = 10). The microbiota of frozen and non-frozen samples in untreated and PMA-treated conditions were compared. Overall 4.04 × 10^6^ reads were recorded from 10 × 2 samples (*m* = 2.02 × 10^5^). Sample storage had an insignificant effect on observed Bray–Curtis community similarity between treatment conditions (Table 3), and no separation by PMA-treatment within storage groups was observed (Figure S3). This data further supports Shaw et al. (2016), findings suggesting freeze storage of severely preterm stool samples does not significantly impact the gut microbiota observed with or without PMA-treatment.

**Table 3:** *P*-values associated with ANOSIM analysis comparing Bray Curtis dissimilarity between frozen and non-frozen samples in control and PMA-treated sample conditions.

	**ANOSIM** × **groups (Bray-Curtis)**			
	**Frozen CTRL**	**Frozen PMA**	**Non-frozen CTRL**	**Non-frozen PMA**
Frozen CTRL		>0.1	<0.01	<0.01
Frozen PMA	>0.1		<0.01	<0.01
Non-frozen CTRL	<0.01	<0.01		>0.5
Non-frozen PMA	<0.01	<0.01	>0.5	

## Discussion

The gut microbiota of significantly preterm infants held within the neonatal intensive care unit has been previously identified to be extremely changeable (Koenig et al., 2011; Bergstrom et al., 2014). Microbial communities colonising this biotope are challenged by frequent antibiotic intervention (Craft et al., 2000), administration of probiotics (AlFaleh and Anabrees, 2014), fluctuating pH due to the use of proton pump inhibitors (Omari et al., 2007, 2009), and gut lumen and immune system maturation (Israel, 1994; Levy, 2007; Strunk et al., 2011). All of these factors complex the first months of a newborn infant's life, thereby it is considered the most unstable with respect to microbiota composition. In order for clinicians to accurately assess the requirement for, and effect of, intervention strategies on infant microbial populations the analysis techniques used must be able to reliably quantify unbiased and viable microbiotas.

Currently techniques either cannot provide results within a short turnaround time at a sufficient phylogenetic resolution to assess the diversity in the gut (bacterial culture), or fail to differentiate viable from non-viable community members (Q-PCR). While RNA sequencing enables exclusive identification of genes actively transcribed by viable cells there are downstream issues regarding storage and contaminating RNAses (Zheng et al., 1996) RNA samples require collection in an RNA preservative (Mutter et al., 2004) which is not always possible in the clinic. Furthermore, use of DNA in combination with PMA eliminates the need for reverse transcription of sequences prior to analysis.

Nocker and Camper (2009), have previously shown PMA-treatment excludes DNA from non-viable cells. This study builds on those results by illustrating PMA-treatment of frozen preterm infant stool alters observable microbiota structure and diversity following paired end targeted 16S rRNA gene sequencing. This would suggest inclusion of non-viable community members during preterm infant stool microbiota analysis introduces a bias. Additionally, DNA from non-viable cells can have significant impact on individual taxa quantification. We propose it may be necessary to employ the use of PMA as a tool for NVCE in 16S rRNA gene sequencing based microbiota analysis. Effects of PMA NVCE should not be attributed to cell death during storage as no difference in PMA effect was observed between frozen and fresh stool samples. It is probable that the changes in abundance illustrated by PMA NVCE are caused by antibiotic, probiotic, or other clinical interventions however further study is required to confirm this.

Importantly, this study illustrates that the presence of particular, clinically relevant taxa may be either over-represented (*Bifidobacterium*), or under-represented (*Clostridium, Staphylococcus*), in the absence of PMA-treatment. This is most probably due to the suppression of DNA sequence reads from rare taxa by dominant taxa as illustrated by the reduced bacterial diversity and presence of rare taxa observed in PMA-treated samples.

These findings are of particular relevance in the gut microbiota of the preterm infants analysed in this study due to administration of probiotic supplements. While *Bifidobacterium* remained ubiquitous and abundant across samples in both treatment conditions it has been shown in several studies (Alander et al., 2001; Charbonneau et al., 2013; Rattanaprasert et al., 2014) that administered probiotic strains often fail to engraft long-term. This would make PMA treatment extremely important for analysis of future intervention trials of this manner. Maldonado-Gómez María et al. (2016), showed that presence of phylogenetically or functionally similar keystone species can prevent engraftment of probiotic strains. The results of this study suggest *Bifidobacteria* within the probiotics may not maintain viability throughout the entire GI tract; a further possible reason for this failed engraftment. We demonstrate persistent DNA from non-viable *Bifidobacteria* may conceal the presence of less abundant, transient colonisers with the potential to confound clinical inferences drawn from 16S rRNA gene sequencing data. Further work should compare the functional profiles of the probiotic *Bifidobacterium* strain in Infloran® and the bacterial metagenomes of patients administered the supplement as well as investigating the community engraftment potential of the specific probiotic strains administered to patients enrolled in this study.

This study has deliberately selected a pair of twins with good longitudinal sampling to evaluate the effect of PMA treatment on observable microbiota members, however the number of actual samples (*n* = 16), is relatively small and recruitment purely convenience based. In lieu of the individual taxa for which significant changes in abundance are observed in this study may not be replicated in repeated studies, dependant on viable and non-viable taxa abundances. Specifically, *Bifidobacterium* may not necessarily be observed at significantly altered abundances in microbiota of patients not receiving probiotics or in patients with a greater engraftment potential. Given the key role of *Bifidobacteria* in preterm gut health, this requires further exploration. We stress that use of PMA need not be limited to that of preterm infant stool but could be applied to any unstable environment where clinical microbiota intervention is employed or in which abundance of community members may be regularly changeable. Further studies may wish to explore the use of such techniques in these environments.

Consideration should be granted that the use of a nested PCR technique represents a potential source of amplification bias in populations of low bacterial load (Yu et al., 2015). In contrast, Fan et al. (2009), have demonstrated that use of a 25 cycle nested PCR does not significantly affect observable bacterial communities. Moreover, nested PCR was employed in this study to increase the inhibitory capacity of PMA on chelated DNA, rather than increase identifiable sequences.

One possible reason for the widespread disregard of PMA use for NVCE could be the specification of non-viable cells solely as membrane-compromised cells using this method. Contreras et al. (2011), describe membrane integrity as a “conservative parameter” for viability identification, explaining inability to culture bacteria occurs sooner than membrane denaturation in heat-killed cells. We propose conservative NVCE is more appealing in this clinical context than nihil NVCE, in which non-viable DNA persists and can bias results or exaggerated NVCE, where community members may be excluded from analysis while still viable.

This study represents the first time PMA-treatment has been combined with paired end targeted 16S rRNA gene sequence analysis of a gut microbiota using the methods described by Kozich et al. (2013). Future research should focus on validation of this method of analysis in a larger sample cohort to include greater inter-sample microbiota variation. Analysis of probiotic and commensal bacterial viability throughout the preterm infant GI tract would be another logical progression from this work.

## Ethics statement

This study was carried out in accordance with the recommendations of “NRES Committee North East – Newcastle & North Tyneside 2” with written informed consent from all subjects. All subjects gave written informed consent in accordance with the Declaration of Helsinki. The protocol was approved by the “NRES Committee North East – Newcastle & North Tyneside 2.”

## Author contributions

GY, Cv, and CL conceived the study. NE and JB collected samples and clinical data. GY designed the study and performed the experiments. DS ran the sequencing. GY, Cv, ES, and SC analysed the data. GY, DS, and Cv wrote the paper. All authors proof read and approved the paper prior to submission.

### Conflict of interest statement

The authors declare that the research was conducted in the absence of any commercial or financial relationships that could be construed as a potential conflict of interest.
