# Supplementary material for: Reducing Viability Bias in Analysis of Gut Microbiota in Preterm Infants at Risk of NEC and Sepsis
Source: Front Cell Infect Microbiol. 2017 Jun 6;7:237. doi: 10.3389/fcimb.2017.00237 (PMC5459914; doi:10.3389/fcimb.2017.00237)
Supplement: Supplementary file 1 [file DataSheet1.DOCX]

**Supplementary materials**

*Figure S1*

Figure S1 illustrates the total number of reads per sample following removal of sequences not passing the in-built illumina phred score quality filter (≥Q30), and quality filtering in Mothur.


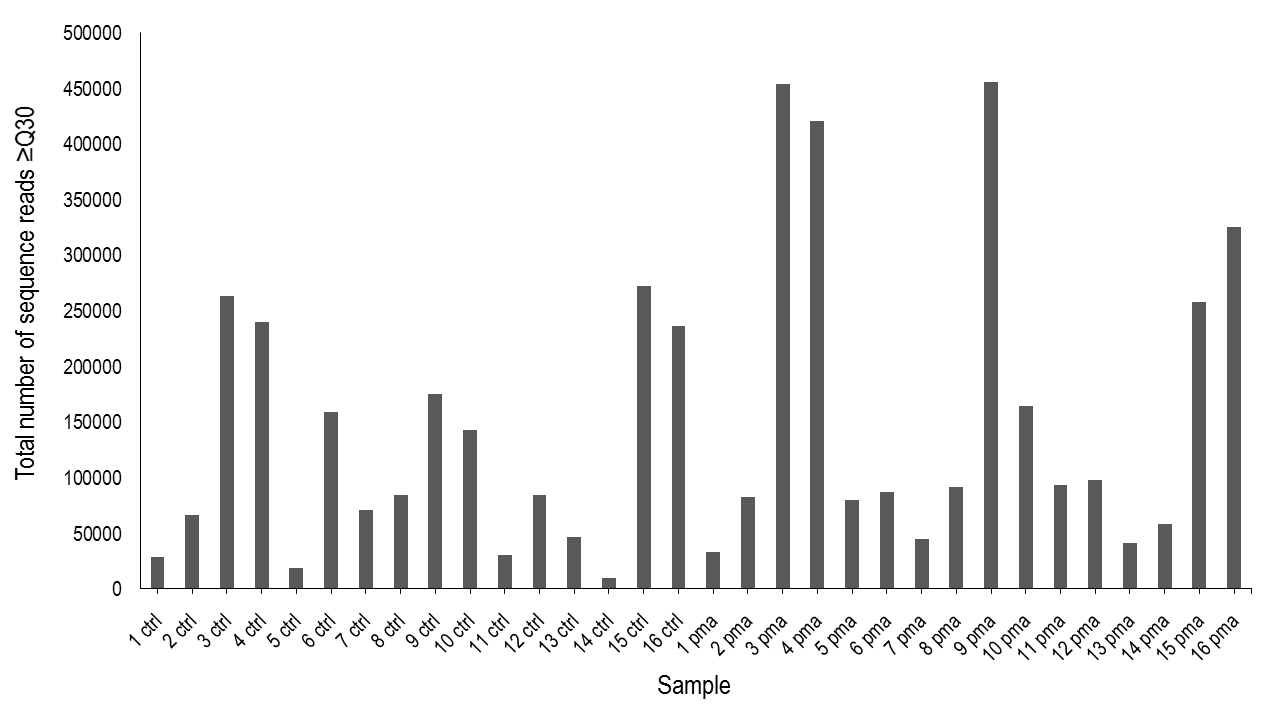


*Figure S2*



Figure S2 shows principle coordinates analysis of Bray-Curtis dissimilarity between both control and PMA-treatment conditions of all 16 sample communities, coloured by sample. Non-PMA-treated samples are circle, PMA-treated conditions are triangle. Lines are extended between conditions of the same sample.

*Figure S3*

Figure S3 shows a boxplot representation of the results of Bray Curtis dissimilarity based ANOSIM analysis between frozen and non-frozen samples under both control and PMA-treated conditions.
